# Intragastric Balloon Treatment Enhances Weight Maintenance Adjunct to Low‐Energy Diet and Group‐Based Cognitive Behavioural Therapy: A Randomized Controlled Trial

**DOI:** 10.1111/dom.70865

**Published:** 2026-06-03

**Authors:** Marije Galavazi, Qays Shahed, Hugo Hesser, Yang Cao, Stefan Jansson, Michiel van Nieuwenhoven, Johan Jendle

**Affiliations:** ^1^ Department of Internal Medicine, Faculty of Medicine and Health Örebro University Örebro Sweden; ^2^ School of Behavioural, Social and Legal Sciences, Center for Health and Medical Psychology Örebro University Örebro Sweden; ^3^ Clinical Epidemiology and Biostatistics, School of Medical Sciences, Faculty of Medicine and Health Örebro University Örebro Sweden; ^4^ Faculty of Medicine and Health University Health Care Research Center, Örebro University Örebro Sweden; ^5^ Division of Gastroenterology, Department of Internal Medicine, Faculty of Medicine and Health Örebro University Örebro Sweden; ^6^ School of Medical Sciences Örebro University Örebro Sweden

**Keywords:** efficacy, health‐related quality of life, obesity treatment, randomized controlled trial, safety

## Abstract

**Aims:**

Low‐energy diet (LED) plus cognitive‐behavioural therapy (CBT) produces substantial short‐term weight loss in obesity, but long‐term maintenance remains difficult. We evaluated whether adding intragastric balloon (IGB) treatment to group‐based CBT improves weight maintenance after initial weight loss using LED.

**Materials and Methods:**

In this open‐label randomized controlled trial, adults aged 30–65 years with BMI 32.5–45 kg/m^2^ underwent a 6‐month run‐in period combining LED and group‐based CBT and were subsequently randomized to CBT and 1‐year IGB or CBT alone. The primary endpoint was weight change in percent between randomization (6 months from enrollment) and 18 months (from enrollment), compared to total body weight (TBW) at enrollment. Secondary outcomes included the proportion of participants achieving weight loss thresholds, metabolic parameters and health‐related quality of life (HRQoL).

**Results:**

Of 126 enrolled (mean 43.7 years; BMI 38.0 kg/m^2^), 107 were randomized 6 months after enrollment (IGB *n* = 51; CBT *n* = 56) and lost −18.7% (SE: 0.64) of TBW. At 18 months, the mean weight change from randomization was 5.2% with IGB versus 10.1% with CBT (*p* < 0.001). A significantly greater proportion of IGB recipients achieved ≥ 5% (84.3% vs. 63.6%; *p* = 0.021) and ≥ 10% (70.6% vs. 45.5%; *p* = 0.013) weight loss after 18 months, compared to the CBT group. Improvements in metabolic parameters and HRQoL showed no between‐group differences.

**Conclusions:**

As an adjunct to group‐based CBT and after LED‐induced weight loss, IGB treatment provided a modest advantage in weight maintenance for up to 18 months, although safety concerns remain and long‐term studies are warranted.

## 1 Introduction

Obesity reduces health‐related quality of life (HRQoL) and elevates the risk for type 2 diabetes, cardiovascular disease, metabolic dysfunction‐associated steatotic liver disease (MASLD), sleep apnea and several types of cancer, where the risk increases with increasing BMI [1, 2, 3, 4, 5]. Globally, obesity prevalence and burden have risen for decades [6]. In Sweden, 18.2% of adults aged 16–84 years had a BMI of 30 kg/m^2^ or more in 2024 [7].

More than 5% of total body weight (TBW) reduction significantly improves metabolic health and HRQoL [8, 9]. However, sustaining such weight loss in the long‐term (≥ 12 months) remains challenging [10]. Major barriers to sustained weight loss include metabolic and neuroendocrine adaptations, making the body prone to gain weight after weight loss [10, 11].

Current obesity management emphasizes lifestyle interventions that integrate dietary and eating behaviour modifications, physical activity and behavioural strategies to promote sustained health improvements [12]. Intensive lifestyle interventions can achieve an average weight loss of 7%–8% [13, 14]. However, when further weight loss is required, additional therapeutic approaches are often necessary [15].

Clinical guidelines recommend behavioural, psychological, pharmacological and surgical treatments for obesity, used alone or in combination [2]. Intragastric balloon (IGB) therapies are FDA‐ and EMA‐approved and show short‐term efficacy [16].

Cognitive behavioural therapy (CBT) promotes lifestyle changes that facilitate weight stability after initial weight loss by developing self‐management skills using techniques such as goal‐setting, cognitive restructuring of negative thoughts, problem‐solving, stimulus control and self‐monitoring [17, 18]. Intensive and prolonged CBT in group format has demonstrated superior long‐term weight stability compared to standard care [19].

Low‐energy diets (LEDs) have proven effective for rapid weight loss [20, 21]. In a 2‐year intervention trial involving 55 adults with a BMI > 35 kg/m^2^, a 12‐week liquid LED (800–880 kcal/day) combined with group‐based CBT resulted in a substantial initial weight loss of 18.9% after 6 months [22]. However, a gradual weight gain was observed thereafter, with the average weight reduction from baseline decreasing to 13.7% after 1 year, and 7.2% after 2 years [22].

IGB treatment is widely used for obesity management, but evidence on long‐term efficacy remains limited [16]. The IGB is endoscopically inserted into the stomach, then filled with saline solution or gas and typically remains in place for 6–12 months, whereafter it is endoscopically punctured, emptied and removed [23]. The IGB promotes weight loss through gastric volume restriction, delayed gastric emptying and enhanced satiety [24, 25]. A systematic review reported a pooled weight loss of 7.6%–14.1% following 6 months of IGB treatment and 7.5%–14.0% after 12 months [26]. To our knowledge, the IGB Orbera365 (Apollo Endosurgery Inc., Austin, TX) has not been evaluated with a randomized controlled trial.

### 1.1 Research Question

The primary aim of this study was to investigate the effects on weight maintenance when adding 12 months IGB treatment to group‐based CBT compared with CBT alone, following an initial 6‐month weight loss phase combining LED and group‐based CBT. The primary hypothesis is that adjunct IGB treatment will lead to less weight regain after initial weight loss compared to without IGB treatment.

## 2 Materials and Methods

### 2.1 Study Design

This was an open‐label, randomized controlled clinical trial (RCT), approved by the Swedish Ethical Review Authority (Main approval: Dnr. 2019‐04756. Amendments: Dnr. 2020‐06552, 2022‐01388‐02 and 2023‐01458‐02). All participants provided written informed consent prior to inclusion in the study. Clinical trial number: NCT04230655.

### 2.2 Participants

Adults aged 30–65 years with a BMI between 32.5–45 kg/m^2^ were eligible for participation. Key exclusion criteria included recent weight loss treatments within 3 months prior to enrollment and any contraindications to IGB treatment. (Table S1).

### 2.3 Procedures

#### 2.3.1 Run‐In Period

Following enrollment, participants underwent a 6‐month run‐in period combining LED and group‐based CBT.

#### 2.3.2 Randomization

**FIGURE 1:**
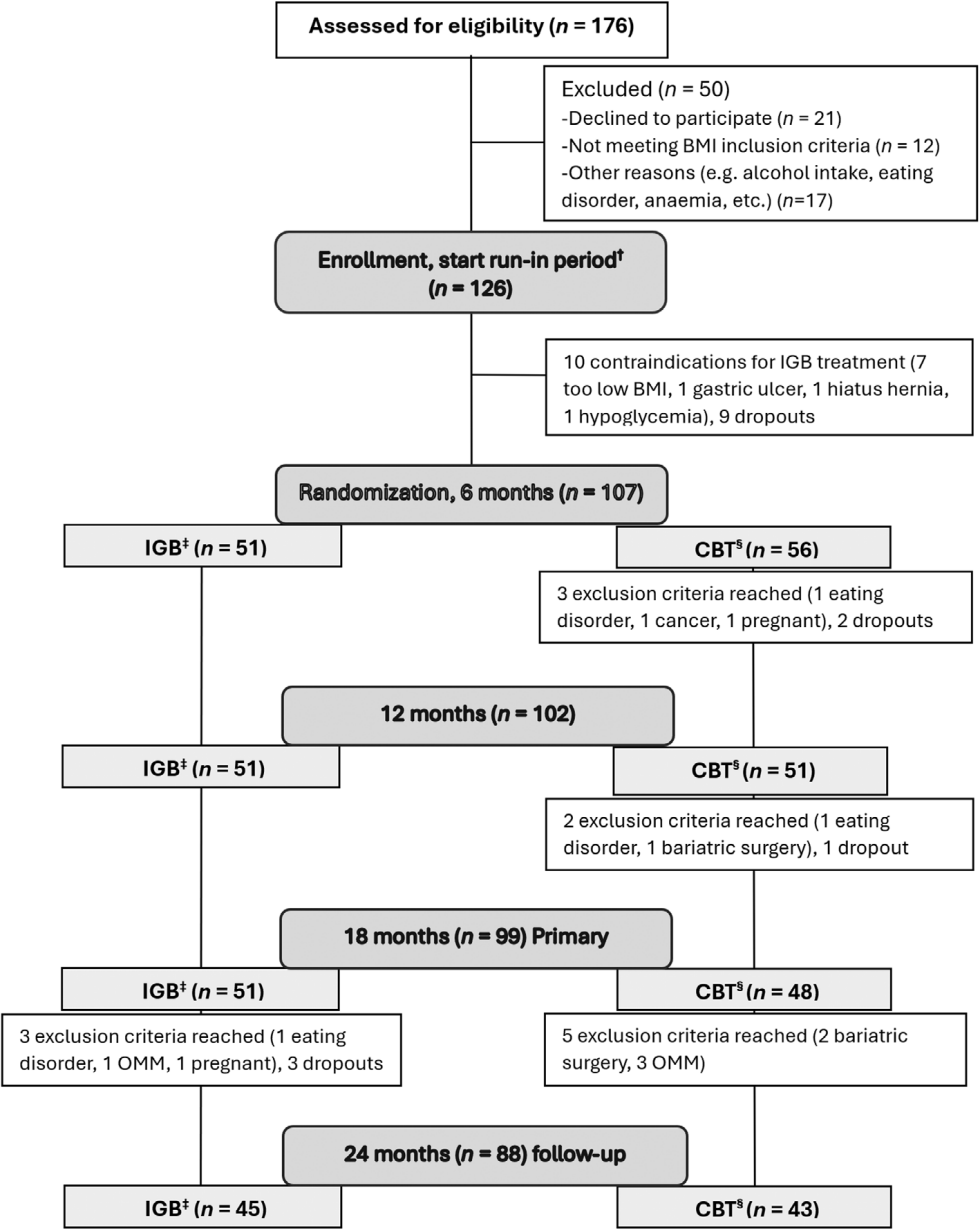
Study flow chart. Abbreviations: BMI, body mass index; CBT, cognitive‐behavioural therapy; IGB, intragastric balloon; OMM, obesity management medication. †6‐month run‐in: All participants received a combination of group‐based CBT and low‐energy diet. ‡6 months continued group‐based CBT and a maximum of 12 months IGB treatment. §6 months continued group‐based CBT.

Participants were randomized using block randomization, 1:1 ratio, generated within the eCRF. Randomization remained double‐blinded during the 6‐month run‐in period. Upon completion of this phase, the randomization codes were disclosed, referred to as the randomization time point. Participants were allocated to the intervention group, IGB treatment plus group‐based CBT sessions (IGB group), or the control group, group‐based CBT sessions only (CBT group). Participants who met any exclusion criteria at the time of randomization (before IGB insertion) were excluded from further analysis (Figure 1).

#### 2.3.3 CBT

All participants were offered 14 sessions with 2.5 h group‐based CBT, once every 4 weeks, over a one‐year period, at the Obesity Unit, Örebro University Hospital, Sweden. The sessions were held by two group leaders (dietitian and/or psychologist and/or physiotherapist).

The structured CBT program used CBT techniques like goal setting, self‐monitoring, identifying and reframing of negative thoughts, and relapse prevention. Key themes addressed during sessions included eating behaviour, nutrition, dietary habits, physical activity, sleep, realistic goal setting and strategies for coping with stress and setbacks. Participants were recommended to increase physical activity with levels depending on their individual ability, if possible, up to at least 150 min a week [2].

#### 2.3.4 LED

During the initial 12 weeks of the run‐in period, participants followed a strict LED providing 936–963 kcal/day using liquid meal replacements (Modifast, Impolin AB, Täby, Sweden) followed by a 12‐week gradual phasing out to regular meals. Subsequently, all participants were advised to adhere to the Nordic Nutrition Recommendations (NNR), with a reduced energy intake of 1400–1600 kcal/day [27]. These recommendations were valid throughout the treatment for both the CBT and the IGB group.

#### 2.3.5 IGB: After Randomization

Participants randomized to the IGB group underwent endoscopic placement of an IGB (Orbera365) under propofol sedation. Peri‐ and postprocedural care was offered in accordance with the AGA Clinical Practice Guidelines on IGBs in the management of obesity [23]; a proton‐pump inhibitor was prescribed during the IGB treatment period; antiemetics and antispasmodics were provided if necessary; and immediately following IGB insertion, a gradual reintroduction of solid foods was recommended over subsequent days.

### 2.4 Endpoints

The primary endpoint was the percent change in body weight from randomization (6 months after enrollment) to 18 months after enrollment, expressed relative to TBW at enrollment, in the IGB and the CBT group respectively. Secondary endpoints included the proportion of participants achieving weight loss thresholds of ≥ 5%, ≥ 10% and ≥ 15% reduction in TBW from enrollment to 12 and 18 months, respectively.

Descriptive endpoints included: TBW, waist circumference, metabolic parameters, and HRQoL, measured at enrollment, randomization and 12 months (after enrollment).

At 24 months, safety assessments and a follow‐up of weight were conducted.

### 2.5 Assessments

Body weight was measured using a calibrated scale (BC‐420MA, Tanita Europe BV, Amsterdam, the Netherlands).

At enrollment, 6‐ and 12 months after enrollment, both fasting metabolic parameters and HRQoL were assessed. HRQoL was evaluated using the RAND‐36 questionnaire, a publicly available version of the Medical Outcome Study Short Form‐36 (MOS‐SF36) [28]. Overall well‐being was assessed by aggregating the RAND‐36 domains into two composite scores: the Mental Composite Score (MCS) and the Physical Composite Score (PCS) [29] where higher scores indicate better HRQoL.

As safety assessment, participants were routinely asked to report any AEs at each study visit and medical records were reviewed for additional safety monitoring.

### 2.6 Sample Size Calculation

The sample size was calculated to detect a mean difference of 5% in TBW change between the IGB and CBT groups at 18 months after enrollment. Assuming a standard deviation of 8% for percent TBW change, a two‐sided *α* level of 0.05, and 80% statistical power, 88 participants (44 per group) were required. To account for an anticipated dropout rate of approximately 30% during the 6‐month run‐in period and subsequent follow‐up, a total of 126 participants were planned for enrollment at study initiation.

### 2.7 Statistical Analysis

Efficacy analyses were conducted according to the intention‐to‐treat principle, including all randomized participants who remained eligible at the time of randomization, regardless of the duration of IGB treatment, attendance at group sessions after randomization or early discontinuation. Safety analyses included all participants who initiated the allocated intervention after randomization.

The primary analysis targeted the prespecified estimand of the between‐group difference in weight regain from randomization to 18 months after enrollment, expressed as percentage of body weight at enrollment. Because randomization occurred after a common 6‐month run‐in period, the randomization time point was considered the baseline for the randomized treatment comparison. Accordingly, the primary endpoint was analysed as a direct between‐group comparison of change from randomization to 18 months. Analyses at other time points were considered secondary and descriptive.

Independent samples *t*‐tests were used to compare continuous variables between treatment groups at relevant time points (baseline, 6‐, 12‐, 18‐ and 24‐months). Data are presented in mean and standard error (SE). Categorical variables, such as the proportion of subjects achieving weight loss thresholds (≥ 5%, ≥ 10% or ≥ 15% TBW reduction) at 12 and 18 months after enrollment, were compared using Pearson's Chi‐Square test.

Missing data were handled under a missing‐at‐random assumption using multiple imputation with 20 imputed datasets, considering the relatively small sample size and missing rates as high as 18.7% in some variables. Imputed variables included body weight, BMI, waist circumference, blood pressure, HbA1c, homeostatic model assessment for insulin resistance (HOMA‐IR), fasting serum lipids and RAND‐36 mental and physical composite scores. Estimates from each imputed dataset were then combined using Rubin's rules [30].

Four sensitivity analyses for the primary endpoint were conducted: (1) a completers‐only analysis using observed 18‐month weight data without imputation in all randomized participants with available outcome data; (2) a per‐protocol analysis including only participants who had available weight data at 18 months after enrollment and for participants in the IGB group, who completed at least 6 months of IGB treatment, under a missing‐at‐random assumption using multiple imputation with 20 imputed datasets; (3) a supportive mixed‐effects model analysis was performed for repeated measures of post‐randomization percent change of body weight, following multiple imputation of missing values (*m* = 20 imputations) under missing at random (MAR) assumption; (4) the fourth sensitivity analysis was performed using a mixed effects model also with multiple imputed data under missing not at random (MNAR) assumption, in which a delta‐adjusted pattern‐mixture approach was used with missing weight regains imputed with +1%, +2.5%, +5% TBW for IGB missing cases and 0%, −1%, −2.5% TBW for CBT missing cases at 12, 18 and 24 months, respectively, to reflect less favourable assumptions for the IGB group and more favourable assumptions for the CBT group.

All statistical tests were two‐sided, and a *p* value < 0.05 was considered statistically significant. Because secondary and exploratory outcomes were not adjusted for multiple comparisons, these findings should be interpreted as exploratory. Analyses were performed using IBM SPSS Statistics, version 25 (IBM Corp).

## 3 Results

### 3.1 Participants

An initial telephone screening was done with 421 individuals who had expressed interest in participating in the study. 176 individuals attended a screening visit, and 126 participants fulfilled the in‐ and exclusion criteria and were enrolled in the study (Figure 1). The study population was divided into eight treatment groups, with two groups starting simultaneously. Baseline characteristics are presented in Table 1.

**TABLE 1:** Characteristics of all participants at enrollment and at randomization.

Characteristics in mean (SE) unless otherwise stated	Start of run‐in (*n* = 126)	Change after 6‐month run‐in (*n* = 107)	At randomization (6 months)	
			IGB (*n* = 51)	CBT (*n* = 56)
Age, years	43.7 (0.76)		44.1 (1.25)	43.3 (1.11)
Women, *n* (%)	112 (88.9)		46 (90.2)	52 (92.9)
Height, cm	168.0 (0.73)		166.0 (1.11)	168.6 (1.00)
Weight, kg	107.6 (1.34)	−20.2 (0.77)	85.9 (1.87)	88.4 (1.71)
Weight change, %		−18.7 (0.64)	−18.5 (0.95)	−18.9 (0.87)
BMI, kg/m^2^	38.0 (0.33)	−7.2 (0.26)	31.2 (0.63)	31.0 (0.46)
Waist circumference, cm	116.4 (0.89)	−17.5 (0.79)	98.4 (1.57)	99.5 (1.50)
Blood pressure, mmHg				
Systolic	130.2 (1.33)	−8.8 (1.44)	121.3 (1.80)	121.3 (1.95)
Diastolic	78.2 (0.96)	−5.3 (1.05)	73.0 (1.13)	73.6 (1.13)
HbA1c, mmol/mol	37.8 (0.43)	−2.9 (0.34)	35.1 (0.50)	34.5 (0.37)
HOMA‐IR	4.3 (0.23)	−2.2 (0.17)	2.0 (0.13)	2.1 (0.15)
Fasting serum lipids, mmol/L				
Total cholesterol	5.0 (0.08)	−0.4 (0.06)	4.5 (0.12)	4.6 (0.12)
HDL‐cholesterol	1.3 (0.03)	0.02 (0.02)	1.4 (0.05)	1.3 (0.04)
LDL‐cholesterol	3.5 (0.08)	−0.5 (0.06)	2.9 (0.11)	3.1 (0.12)
Triglycerides	1.5 (0.08)	−0.4 (0.06)	1.0 (0.05)	1.0 (0.06)
RAND‐36 unweighted MCS	60.6 (1.81)	13.6 (1.97)	75.2 (2.91)	73.8 (2.78)
RAND‐36 unweighted PCS	62.7 (1.74)	18.1 (1.90)	80.5 (2.57)	81.0 (2.31)

*Note:* Statistical analyses were performed on pooled imputed data after multiple imputation (20 datasets).

Abbreviations: BMI, body mass index; HDL, high‐density lipoprotein; HOMA‐IR, homeostatic model assessment for insulin resistance; IGB, intragastric balloon; LDL, low‐density lipoprotein; MCS, mental composite score; PCS, physical composite score.

Nine participants dropped out during the run‐in period and 10 participants, eight in the IGB and two in the CBT group, were excluded from analysis due to contraindications for IGB treatment identified at the randomization time point (Figure 1).

Following the described intention‐to‐treat principle, 107 participants were included in the analysis with *n* = 51 in the IGB and *n* = 56 in the CBT group. At randomization, the IGB and CBT groups were well‐matched (Table 1).

### 3.2 Changes at 6 Months After Enrollment

After the run‐in period, participants achieved a mean TBW reduction of 18.7% (SE: 0.64). The mean TBW change in the IGB group was −18.5% (SE: 0.95), and in the CBT group −18.9% (SE: 0.87) (*p* = 0.731), demonstrating comparable reductions across both treatment arms. Both the IGB and CBT groups showed significant improvements in BMI, waist circumference, blood pressure, HbA1c, HOMA‐IR, serum lipids and HRQoL from enrollment to randomization, without significant differences between the groups (Table 1).

### 3.3 Changes at 12 and 18 Months After Enrollment

The primary endpoint showed a difference between the groups with a weight regain in the IGB group of 5.2% (SE: 1.00) 18 months after enrollment versus 10.1% (SE: 0.92) in the CBT group (*p* < 0.001). Sensitivity analysis 3, using a mixed‐effects model, revealed a statistically significant main effect of intervention on weight change after randomization. Participants in the IGB group had less weight regain at 18 months (−5.47; 95% CI: −8.24, −2.70; *p* < 0.001) compared to the CBT group, after adjustment for age, sex, height and weight at randomization (6 months) (Table S2). Regarding the intervention‐by‐time interaction, the sensitivity analysis indicated a statistically significant interaction between time and intervention, which resulted in less weight regain (−2.14%; 95% CI: −3.83%, −0.46%; *p* = 0.013) in the IGB group at 18 months. In other words, while weight rose over time in both groups, the rate of increase was attenuated in the IGB group at 18 months. However, at 24 months, the interaction term was not statistically significant, suggesting that the weight regain trend between groups was no longer statistically distinguishable at the end of follow‐up (Figure S1 and Table S2).

**FIGURE 2:**
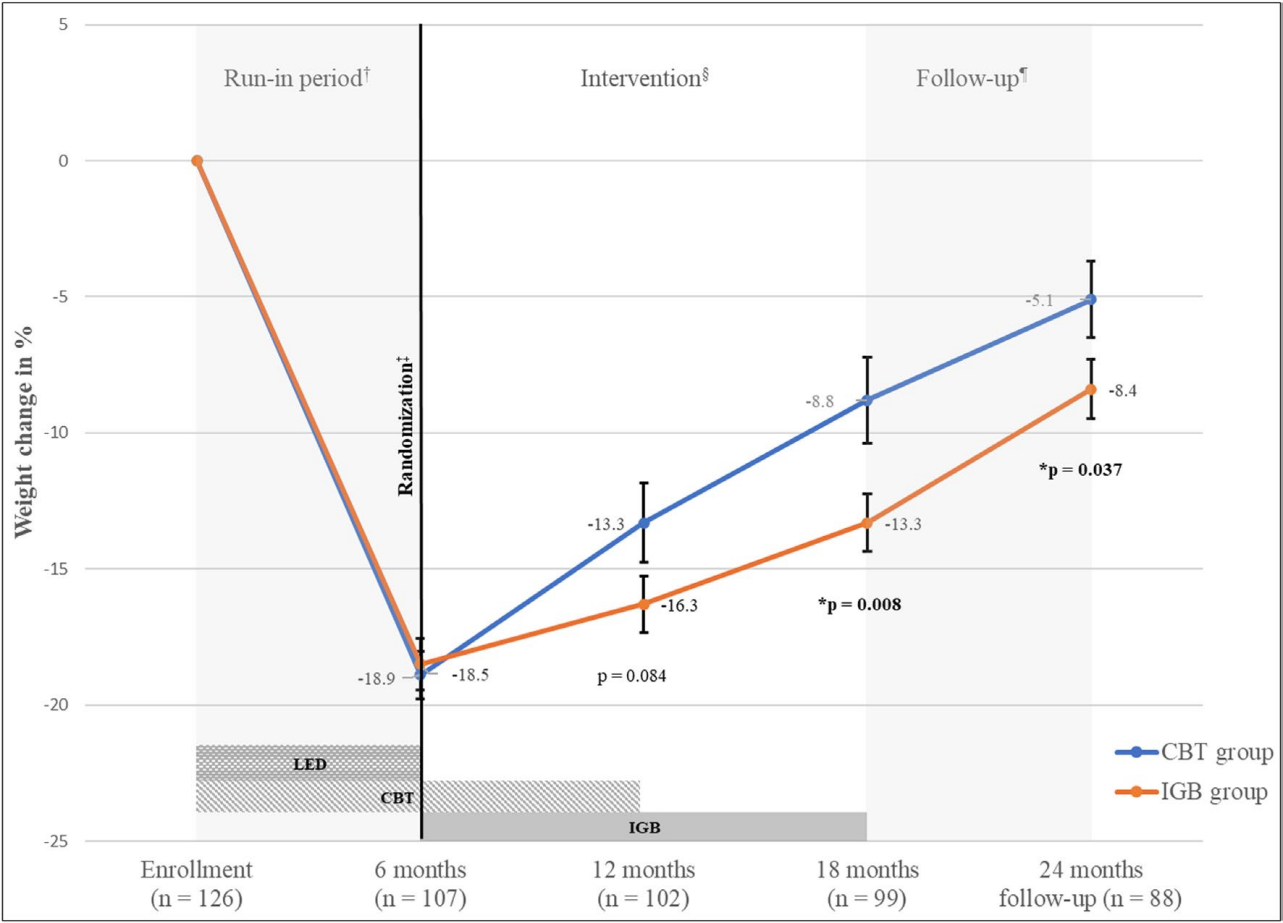
Mean change in body weight (%) from TBW for the entire study period: Enrollment (*n* = 126, mean weight 107.6 kg), randomization at 6 months (*n* = 107), 12 months (*n* = 102), 18 months (primary endpoint, *n* = 99) and at 24‐months follow‐up (*n* = 88). Abbreviations: BMI, body mass index; CBT (striped pattern), cognitive‐behavioural therapy; LED (checked pattern), low‐energy diet; IGB (solid pattern), intragastric balloon; TBW, total body weight. Error bar: 95% confidence interval of SE. †Run‐in period; 6‐month run‐in period: All participants received a combination of group‐based CBT and LED. ‡Randomization: Participants were randomized, ratio 1:1, to either the IGB or CBT group. §Intervention; IGB group received 6 months CBT plus IGB treatment up to 12 months, CBT group received 6 months CBT. ¶Follow‐up; The 12‐month treatment was followed by a 6‐month follow‐up period.

Total weight change at 12 and 18 months after enrollment compared to TBW at enrollment was −16.3% (SE: 1.05) and −13.3% (SE: 1.07) in the IGB group, and −13.3% (SE 1.39) and −8.8% (SE: 1.30) in the CBT group, respectively (*p* = 0.084 and *p* = 0.008, respectively) (Figure 2). From randomization to 12 and 18 months after enrollment, mean BMI increased from 31.2 to 32.0 (SE: 0.62) and 33.1 kg/m^2^ (SE: 0.63) in the IGB group and in the CBT group from 31.0 to 33.1 (SE: 0.55) and 34.8 kg/m^2^ (SE: 0.55) (between‐group difference; *p* = 0.165 and *p* = 0.053, respectively) (Table 2).

**TABLE 2:** Endpoints at 12, 18 and 24 months.

Descriptive endpoints at 12 months				
In mean (SE) unless otherwise stated	IGB (*n* = 51)	CBT (*n* = 51)	Mean difference (95% CI of difference)	*p*
Weight change from randomization compared to TBW at enrollment, %	2.2 (0.70)	5.7 (0.89)	3.5 (1.25 to 5.72)	0.002*
Total body weight, kg	88.2 (0.62)	94.6 (2.12)	6.4 (0.75 to 12.12)	0.027*
Weight change from randomization, kg	2.3 (0.74)	6.0 (0.85)	3.7 (1.50 to 6.00)	0.001*
Weight change from randomization, %	2.8 (0.87)	6.8 (0.99)	4.0 (1.39 to 6.60)	0.003*
Regain proportion of lost weight, %	7.2 (0.08)	41.0 (0.06)	33.8 (14.24 to 53.26)	**0.001**
Weight change from enrollment, %	−16.3 (1.05)	−13.3 (1.39)	3.04 (−0.41 to 6.49)	0.084
BMI, kg/m^2^	32.0 (0.62)	33.1 (0.55)	1.1 (−0.47 to 2.76)	0.165
Waist circumference, cm	101.9 (1.67)	105.1 (1.82)	3.2 (−1.65 to 8.12)	0.194
Blood pressure, mmHg				
Systolic	123.1 (1.98)	125.17 (1.78)	2.2 (−3.01 to 7.41)	0.410
Diastolic	75.5 (1.41)	77.5 (2.93)	1.9 (−5.36 to 9.24)	0.601
HbA1c, mmol/mol	35.8 (0.60)	35.2 (0.45)	0.6 (−2.2 to 0.9)	0.378
HOMA‐IR	2.4 (0.21)	2.6 (0.24)	0.2 (−2.11 to 0.80)	0.565
Fasting serum lipids, mmol/L				
Total cholesterol	4.7 (0.13)	4.8 (0.13)	0.1 (−0.24 to 0.50)	0.486
HDL‐cholesterol	1.5 (0.06)	1.5 (0.05)	0.1 (−0.23 to 0.07)	0.303
LDL‐cholesterol	2.9 (0.12)	3.1 (0.12)	0.1 (−0.22 to 0.44)	0.511
Triglycerides	1.3 (3.32)	1.5 (3.00)	0.2 (−0.93 to 1.29)	0.749
RAND‐36 unweighted MCS	70.1 (3.37)	63.3 (3.01)	6.7 (−15.57 to 2.12)	0.136
RAND‐36 unweighted PCS	80.0 (2.45)	73.0 (2.94)	7.2 (−14.68 to 0.34)	0.064
Group attendance, no. of sessions attended of 14	10.9 (0.35)	10.7 (0.41)	0.3 (−1.33 to 0.81)	0.630
**Descriptive endpoints at 18 months (other data noted at 12 months was not collected at this time point)**				
	**IGB (*n* = 51)**	**CBT (*n* = 48)**		
Weight change from randomization compared to TBW at enrollment, %	5.2 (1.00)	10.1 (0.92)	4.9 (2.26 to 7.60)	< 0.001*
Total body weight, kg	91.4 (2.01)	99.2 (2.06)	7.8 (2.10 to 13.43)	0.007*
Weight change from randomization, kg	5.5 (1.10)	10.6 (1.03)	5.1 (2.12 to 8.01)	0.001*
Weight change from randomization, %	6.7 (1.28)	12.1 (1.22)	5.4 (1.94 to 8.86)	0.002*
Regain proportion of lost weight, %	22.3 (12.91)	63.3 (7.83)	41.0 (12.06 to 70.00)	0.006*
Weight change from enrollment, %	−13.3 (1.07)	−8.8 (1.30)	4.49 (1.15 to 7.83)	0.008*
BMI, kg/m^2^	33.1 (0.63)	34.8 (0.55)	1.6 (−0.02 to 3.26)	0.053
**Descriptive endpoints at 24 months (other data noted at 12 months was not collected at this time point)**				
	**IGB (*n* = 45)**	**CBT (*n* = 43)**		
Weight change from randomization compared to TBW at enrollment, %	10.1 (1.08)	13.8 (0.86)	3.7 (1.00 to 6.36)	0.007*
Total body weight, kg	96.6 (2.00)	102.9 (2.08)	6.3 (0.62 to 12.02)	0.030*
Weight change from randomization, kg	10.7 (1.25)	14.5 (1.13)	3.4 (0.12 to 6.59)	0.042*
Weight change from randomization, %	13.1 (1.49)	16.4 (1.29)	3.26 (−0.57 to 7.10)	0.095
Regain proportion of lost weight, %	52.7 (9.8)	81.6 (7.81)	29.9 (4.74 to 53.03)	0.019*
Weight change from enrollment, %	−8.4 (1.02)	−5.1 (1.16)	3.2 (0.19 to 6.28)	0.037*
BMI, kg/m^2^	35.0 (0.63)	36.1 (0.58)	1.1 (−0.60 to 2.78)	0.205
Waist circumference, cm	108.7 (1.53)	111.5 (1.67)	2.8 (−1.69 to 7.29)	0.221
Blood pressure, mmHg				
Systolic	128.8 (1.83)	129.8 (2.20)	1.0 (−4.60 to 6.57)	0.729
Diastolic	76.9 (1.29)	76.7 (1.58)	0.3 (−4.24 to 3.70)	0.895
RAND‐36 unweighted MCS	62.0 (4.69)	58.8 (10.05)	−2.8 (−28.58 to 22.97)	0.830
RAND‐36 unweighted PCS	69.8 (3.73)	67.8 (6.21)	−3.3 (−19.57 to 13.01)	0.692

*Note*: *p < 0.05 indicates a significant between‐group difference (in bold).

Abbreviations: BMI, body mass index; CBT, cognitive behavioural therapy; CI, confidence interval; HDL, high‐density lipoprotein; HOMA‐IR, homeostatic model assessment for insulin resistance; IGB, intragastric balloon; LDL, low‐density lipoprotein; MCS, mental composite score; PCS, physical composite score.

The first sensitivity analysis, with a completers‐only analysis using observed 18‐month weight data without imputation in all randomized participants with available outcome data, included 51 IGB and 44 CBT participants. The analysis showed a weight regain in the IGB group of 5.2% (SD: 7.16) and 10.7% (SD: 7.41) in the CBT group (*p* < 0.001).

The second sensitivity analysis (per‐protocol analysis) showed a weight regain of 9.4% (SE: 1.11) in the IGB group (*n* = 44) and 14.3% (SE: 1.22) in the CBT group (*n* = 43) (*p* = 0.003) (Figure S2).

Sensitivity analysis 4, conducted under a MNAR framework with progressively conservative assumptions favouring the CBT group, demonstrated that the superior outcomes observed in the IGB group remained statistically significant at all three follow‐up timepoints. Although the magnitude of the between‐group differences attenuated modestly with delta adjustments, the direction and statistical significance of the treatment effect were preserved across all timepoints (Table S2).

**FIGURE 3:**
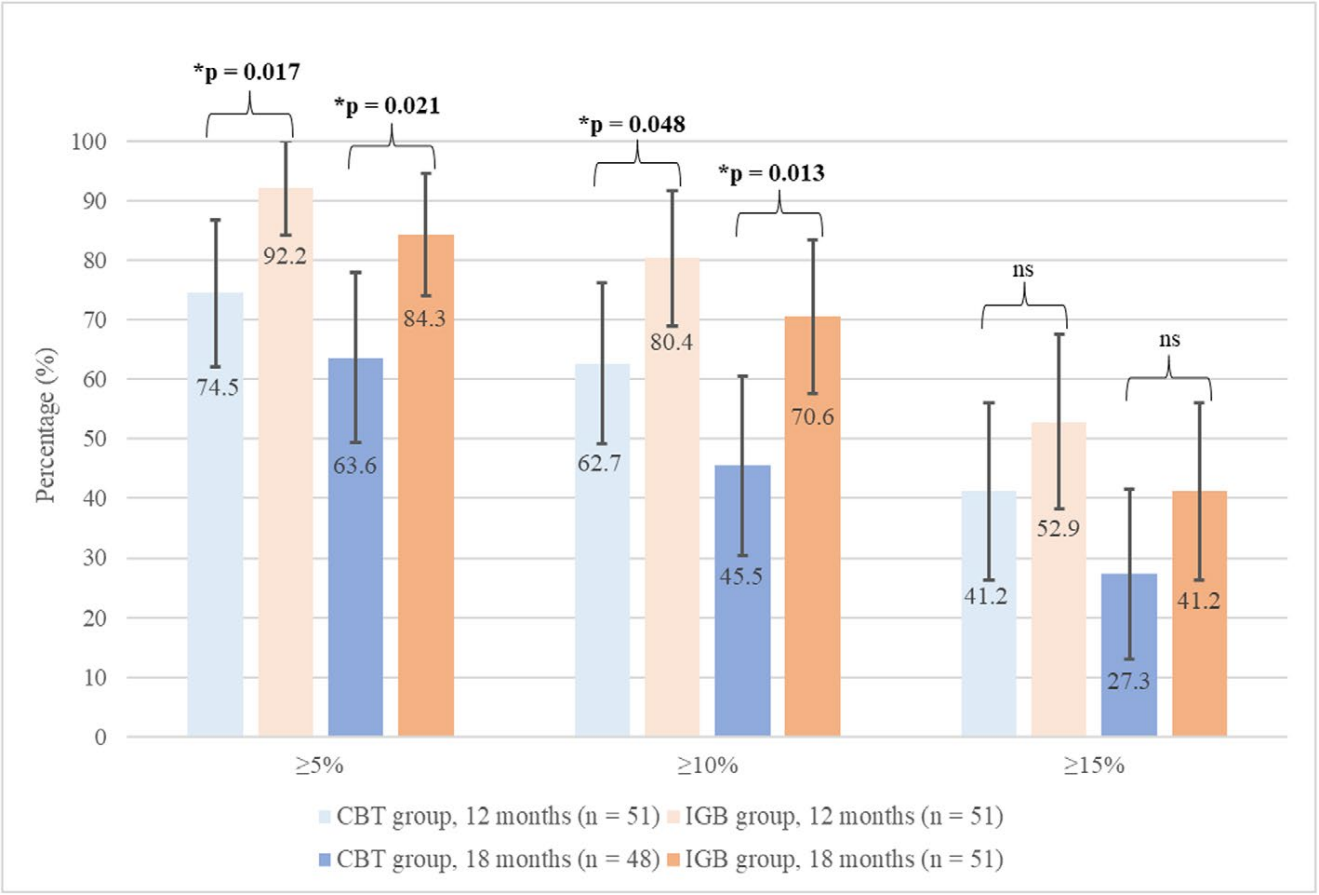
Percentage of participants achieving a threshold of ≥ 5%, ≥ 10% and ≥ 15% weight loss at 12 and 18 months after enrollment, compared to TBW at enrollment. Abbreviations: CBT, cognitive‐behavioural therapy; IGB, intragastric balloon; ns, not significant; TBW, total body weight. The proportion of participants in the IGB and CBT groups achieving ≥ 5%, ≥ 10% and ≥ 15% weight loss at 12 and 18 months after enrollment. *p* < 0.05 indicates a significant between‐group difference. Error bars presented as 95% confidence interval.

The proportion of participants achieving ≥ 5%, ≥ 10% and ≥ 15% TBW loss at 12 and 18 months after enrollment is shown in Figure 3, with data presented based on the observed number of participants in each category. At both 12 and 18 months, a significantly greater proportion of participants in the IGB group achieved a ≥ 5% and ≥ 10% TBW reduction, compared to the CBT group (12 months: *p* = 0.017 and *p* = 0.048; 18 months: *p* = 0.021 and *p* = 0.013 respectively) (Figure 3). However, no significant differences were observed between groups for ≥ 15% TBW loss at 12 and 18 months (*p* = 0.234 and *p* = 0.156).

There were no statistically significant differences between the IGB and CBT groups from randomization to 12 months after enrollment in waist circumference, blood pressure, HbA1c, HOMA‐IR, fasting serum lipids and group attendance (Table 2).

With respect to the HRQoL between randomization and 12 months after enrollment, both the IGB and CBT showed improvements, but neither the MCS nor the PCS showed significant between‐group differences (*p* = 0.136 and *p* = 0.064, respectively) (Table 2).

### 3.4 Changes at 24 Months After Enrollment

At the 24‐month follow‐up, the weight gain in percent from randomization, as compared to TBW at enrollment, was 10.1% (SE: 1.08) in the IGB group and 13.8% (SE: 0.86) in the CBT group (*p* = 0.007). Total weight change at 24 months after enrollment compared to TBW at enrollment was −8.4% (SE: 1.02) in the IGB group and −5.1% (SE: 1.16) in the CBT group (*p* = 0.037) (Figure 2). BMI at follow‐up increased to 35.0 kg/m^2^ (SE: 0.63) in the IGB group and 36.1 kg/m^2^ (SE: 0.58) in the CBT group (*p* = 0.205) (Table 2).

### 3.5 Safety Aspects

During the run‐in period, AEs primarily included gastrointestinal (GI) symptoms and effects related to caloric restriction and weight loss, with dizziness, fatigue, hair loss and headache (Table S3). GI symptoms were most prevalent during the initial weeks of LED treatment, affecting 62 participants. Three participants discontinued LED the first week due to intolerance, and two experienced serious adverse events (SAEs) with acute appendicitis.

Following randomization, all participants in the IGB group reported at least one GI symptom, compared to five participants in the CBT group (Table S3). Seven (13.7%) required early IGB removal due to intolerance: four within 2 weeks, two at 5 weeks and one at 6 weeks.

During the intervention period between 6 and 18 months after enrollment, 13 participants experienced SAEs (7 IGB; 6 CBT group) (Table S3). In the CBT group, three required a cholecystectomy, two were diagnosed with an eating disorder, and one with breast cancer. In the IGB group, four SAEs required hospitalization due to severe GI symptoms within 2 weeks after IGB insertion. This led to early removal of the IGB in two participants (one due to gastric retention, one due to vomiting associated with suspected viral gastroenteritis) while the remaining two participants were able to continue IGB treatment after receiving rehydration and antiemetic treatment. Additionally, difficulties during IGB removal were encountered in three participants, leading to modification of the removal procedure, including anaesthesia and intubation instead of only propofol sedation, to ensure airway protection during IGB extraction.

During the 6‐month follow‐up period between 18 and 24 months after enrollment, one participant in the IGB group was diagnosed with an eating disorder.

## 4 Discussion

In this RCT, the addition of an IGB reduced weight regain following the initial weight loss achieved through LED and CBT compared to those who received CBT alone. Although the primary analysis assumed MAR, the results were similar in completers‐only analysis and remained robust in MNAR sensitivity analyses, supporting the overall conclusion while acknowledging uncertainty due to differential attrition. Additionally, a greater proportion of participants in the IGB group achieved a ≥ 5% and a ≥ 10% TBW reduction respectively at 18 months, compared to the CBT group. In a sensitivity analysis (per‐protocol analysis), similar results in weight regain after randomization were seen (Figure S1).

Our study demonstrated that participants in the IGB group regained less weight over the year after randomization. However, a between‐group difference in total weight loss of 4.5% is modest considering the expected clinical implication for the patient. Although the between‐group difference was still significant up to 6 months after removal of the IGB, it had decreased to a difference in total weight loss of 3.3%. While a decline in efficacy following balloon removal was expected in our study, the weight results at the 24‐month follow‐up suggest potential prolonged benefits beyond the treatment period compared to the CBT group.

In our study, participants achieved an 18.7% loss of TBW most likely due to the LED‐period as the weight loss is consistent with previous studies using this method [20, 21, 22]. This substantial initial TBW reduction likely triggered known compensatory physiological mechanisms after weight loss that drive the body to regain the weight again [10, 11], potentially reducing the IGB's effectiveness in long‐term weight maintenance. Additionally, prolonged exposure to the IGB may lead to physiological adaptation to it, potentially diminishing its effect on satiety. There is limited data on the effect of IGB treatment on hunger and satiety hormones. Fuller et al. [31] reported a decrease in leptin and stable adiponectin and peptide YY levels during IGB treatment, while studies on ghrelin yield conflicting findings, with Mion et al. [32] observing a decrease and Fuller et al. an increase.

Research on the efficacy and safety of IGBs beyond 6 months remains scarce [16, 26]. To date, only a few studies have evaluated 12‐month treatment with the Orbera365 IGB: one prospective study involving 97 patients reported a TBW loss of 16.2% ± 10.1%, while two retrospective analyses with sample sizes of 61 and 93 patients reported an average TBW loss of 14.7%, where Kozlowska‐Petriczko et al. reported no significant difference in weight loss between 6‐ and 12‐month IGB treatment [33, 34, 35].

Concerns regarding the tolerability and safety of IGB treatment remain valid. GI symptoms were reported by all the study participants in the initial weeks following IGB insertion [23, 36, 37, 38]. In our study, the IGB and the CBT group had 7 and 6 SAE's respectively. Although there was no numeric difference, it should be noted that all SAE's in the IGB group were associated to the IGB treatment.

However, despite a higher incidence of AEs in the IGB group, there were no significant between‐group differences in the MCS and PCS. The early IGB removal rate in this study aligned with removal rates from Jamal et al. [33] (14.4%) and Alhashema et al. [35] (9.7%). A retrospective evaluation of 5874 people with obesity treated with an IGB reported complications in 7.3% (*n* = 430) including early extraction in 6.1% (*n* = 357) [38].

The risk of AEs and SAEs emphasizes the importance of patient education before and medical supervision during IGB treatment to ensure awareness of potential risks and appropriate management strategies. Given the procedure‐related resource use and complication risk profile of IGB treatment, the cost‐effectiveness of an adjunctive strategy that yields modest between‐group differences at later time points remains uncertain. This underpins the need for extended follow‐up in our study cohort to be able to evaluate efficacy and safety over time.

No specific safety concerns emerged during the use of meal replacements, aligning with findings from previous large trials utilizing LED [13, 39], and existing reviews confirm that LEDs are generally well tolerated [20, 21]. Three participants in the CBT group needed a cholecystectomy, likely associated with the weight loss achieved [40]. Two participants required an appendectomy during the run‐in period, but no published data linking appendicitis and LED and/or weight loss was found. Despite concerns about the long‐term effectiveness of LED and CBT, both treatments have demonstrated sustained cardiometabolic benefits even years after completion, supporting their role as safe and effective treatment options [21, 41, 42].

### 4.1 Strengths and Limitations

The primary strength of this study lies in its RCT design. Furthermore, the study achieved a low dropout rate. These factors contribute to the robustness and reliability of the study.

However, certain limitations should be acknowledged. The open‐label design, though ethically necessary, may have introduced bias. Another limitation is the asymmetric treatment duration at the 18‐month assessment (IGB from 6 to 18 months vs. CBT 12 months after enrollment). Originally, the IGB treatment was planned for 6 months to align with the completion of CBT. However, prior to the start of the study, the Orbera365 IGB received CE mark approval for 12 months treatment. Given our hypothesis that prolonged IGB treatment could benefit participants, and following approval by the Swedish Ethical Review Authority, all participants were given the option to extend the IGB treatment from 6 to 12 months. This resulted in the IGB group having ongoing treatment between 12 and 18 months where the CBT group did not, although participants with an IGB in place did not have more planned contact with the therapists during this time than the CBT group, except for extraction of the IGB.

In our study, AEs were actively asked for in the IGB group during the first 2 weeks after IGB insertion, but not in the CBT group, which may have contributed to the higher reported incidence of AEs in the IGB group.

Furthermore, dropout rates were different between groups: 14.3% in the CBT group, and 0% in the IGB group at 18 months after enrollment, which may have influenced the results. This disparity is most likely attributed to IGB participants having a medical indication to at least come for removal of the IGB. This potential attrition bias could affect both treatment effect size and the generalizability of the results.

Factors that may limit generalizability are the drop‐out and exclusion of participants prior to the randomization time point, the inclusion criteria concerning BMI and age, as well as the high proportion of female participants.

The participants with contraindications for IGB treatment, despite the treatment arm, were excluded to be able to answer the research question concerning the effect of additional IGB treatment over the standard of care at the Obesity Unit Örebro University Hospital with 1‐year CBT group treatment and 12‐week LED. In two of the 10 excluded participants due to the contraindication for IGB treatment, the contraindication was first discovered during the gastroscopy before placement of the IGB (1 gastric ulcer, 1 hiatus hernia) (Figure 1). The nine drop‐outs were all lost to follow‐up within 4 months, thus prior to unblinding of the treatment arms, and had the same average age and BMI as those included in the analysis. The result of this pre‐randomization selection could have created an enrichment of the study population and thereby influenced generalizability and external validity of our results.

The threshold of 32.5 kg/m^2^ was chosen to account for expected weight loss during the 6‐month run‐in, with the aim to ensure that BMI at the time of IGB placement would be according to device indications (BMI ≥ 27 kg/m^2^). Younger individuals were excluded due to lower expected treatment adherence, while older individuals were excluded due to the higher potential risk of sarcopenic obesity in this population [43, 44]. A high proportion of female participants is consistently reported in obesity studies. Research suggests that women are more likely than men to both seek and be recommended treatment for obesity [45, 46].

## 5 Conclusions

This study demonstrates that IGB, combined with LED and CBT, could be a modestly effective adjunctive option for weight maintenance for up to 18 months after an initial 6‐month run‐in weight loss period with combined LED and CBT. However, safety concerns with IGB treatment remain valid as AE's are common and occasionalSAE's do occur.

For a few selected individuals not achieving aimed weight loss goals with lifestyle interventions, unable to tolerate appetite‐regulating medications, or seeking non‐surgical options, IGB may serve as an effective treatment [37, 47]. Thus, careful patient selection is important, considering potential contraindications. Due to the common occurrence of AEs, particularly in the initial weeks following IGB insertion, close monitoring is recommended.

Future research should focus on long‐term efficacy and safety as well as optimizing combination treatments to enhance long‐term weight loss, improve metabolic health and HRQoL.

## Author Contributions

All authors contributed to the study conception and design, interpreting the data and critical revision of the paper. Marije Galavazi, Qays Shahed and Michiel van Nieuwenhoven were involved in data collection. Michiel van Nieuwenhoven was responsible for insertion and extraction of the intragastric balloons. Marije Galavazi analysed data and wrote the first draft of the paper.

## Funding

This work was supported by the Region Örebro län (OLL‐886441, OLL‐935216, OLL‐939125, OLL‐964732, OLL‐970429, OLL‐983440), Nyckelfonden (OLL‐886841, OLL‐935201, OLL‐973006), Apollo Endosurgery Inc.

## Conflicts of Interest

The authors disclose the following: Johan Jendle has been a lecturer/member of the scientific advisory boards at the following companies: Abbott, AstraZeneca, Boehringer Ingelheim, Eli Lilly, Medtronic, MyLife, Novo Nordisk, Sanofi, and Tandem. Marije Galavazi has been a lecturer at the following companies: Eli Lilly and Novo Nordisk, as well as local principal investigator in clinical trials for Novo Nordisk and Boehringer Ingelheim. The remaining authors declare no conflicts of interest.

## Supporting information


**Figure S1:** Pooled estimated marginal means of weight change from randomization based on mixed effects model from 20 imputations under missing at random (MAR) assumption, adjusted for age, gender, height, and weight at randomization (6 months). Error bars are presented as 95% confidence interval.

**Figure S2:** Mean change in body weight (%) from TBW for both the main analysis as shown in Figure 1 and a sensitivity analysis including 44 of 51 participants (86.3%) in the IGB group who completed at least 6 months of IGB treatment and had available weight data at 18 months, and 43 of 56 participants (76.8%) in the CBT group with weight data at 18 months.

**Table S1:** Inclusion and exclusion criteria.

**Table S2:** Pooled treatment effects based on mixed effects models from 20 imputations under missing at random (MAR) and missing not at random (MNAR) assumptions.

**Table S3:** Adverse events from enrollment to 18 months.

## Data Availability

Access to the dataset of the present study can be requested from Örebro University (ORU), at registrator@oru.se. ORU as research principal will conduct a confidentiality review to establish whether the legal conditions for data release are satisfied.
