# Supplementary material for: Intragastric Balloon Treatment Enhances Weight Maintenance Adjunct to Low‐Energy Diet and Group‐Based Cognitive Behavioural Therapy: A Randomized Controlled Trial
Source: Diabetes Obes Metab. 2026 Jun 3;28(8):7300–11. doi: 10.1111/dom.70865 (PMC13341412; doi:10.1111/dom.70865)
Supplement: Supplementary file 1 — Figure S1: Pooled estimated marginal means of weight change from randomization based on mixed effects model from 20 imputations under missing at random (MAR) assumption, adjusted for age, gender, height, and weight at randomization (6 months). Error bars are presented as 95% confidence interval. [file DOM-28-7300-s001.docx]

**Supplementary Figure S1:** Pooled estimated marginal means of weight change from randomization based on mixed effects model from 20 imputations under missing at random (MAR) assumption, adjusted for age, gender, height, and weight at randomization (6 months). Error bars are presented as 95% confidence interval.

**
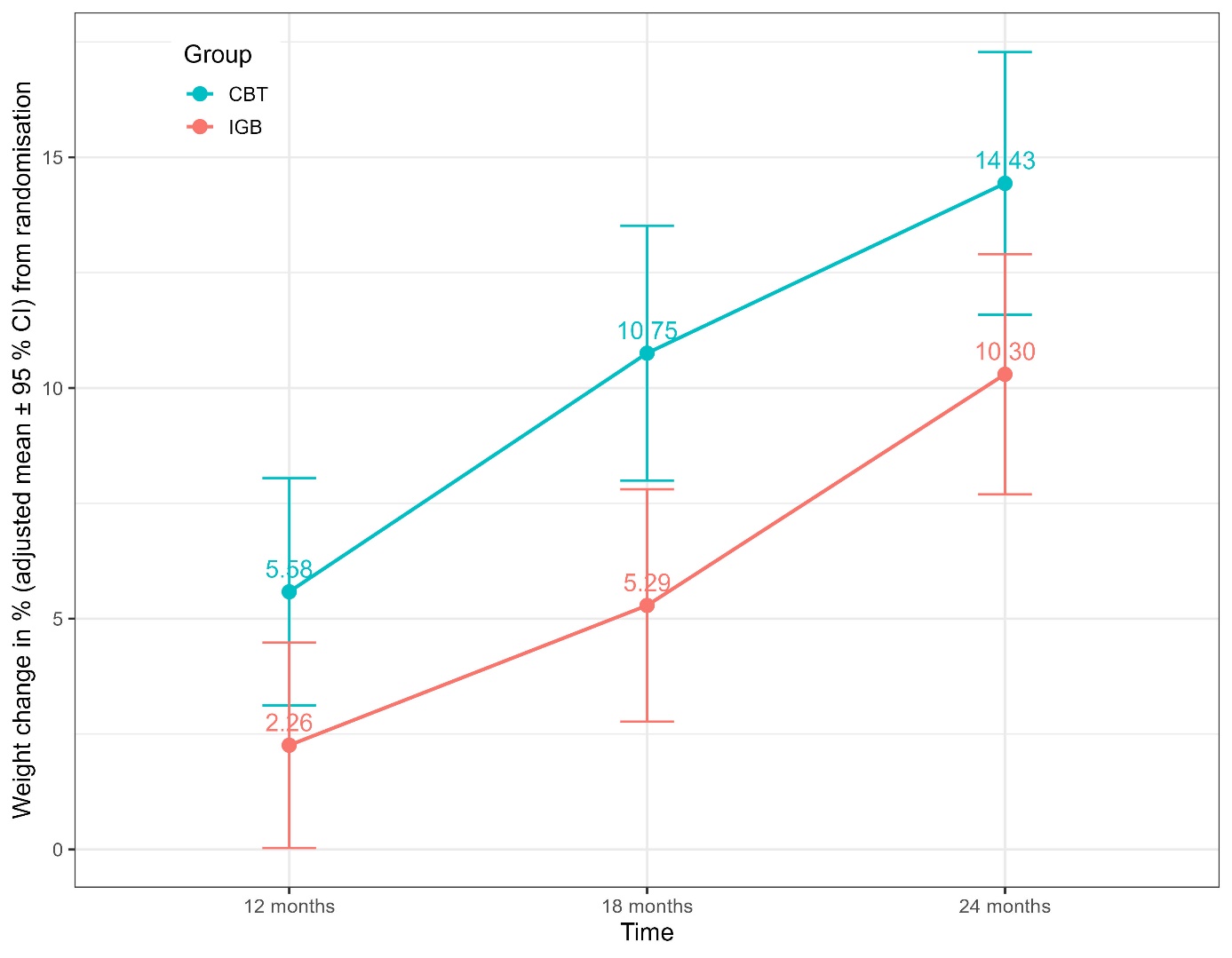
**

Sensitivity analysis of the mixed-effects model for repeated measures, following multiple imputation of missing values (m = 20 imputations), revealed a statistically significant main effect of intervention on weight change after randomization. Participants in the IGB group had less weight regain at 12 months (-3.33%; 95% CI: −5.50%, −1.15%; p = 0.003) compared to the control group, after adjustment for age, sex, height, and weight at randomization (6 months).

Regarding the intervention-by-time interaction, statistically significant further less weight change (−2.14%; 95% CI: −3.83%, −0.46%; p = 0.013) was observed in the IGB group at 18 months. In other words, while weight rose over time in both groups, the rate of increase was attenuated in the intervention group at 18 months, resulting in an overall effect of -5.47% (95% CI: -8.24%, -2.70; p < 0.001). At 24 months, the interaction term was not statistically significant (−0.81%; 95% CI: −3.39%, 1.77%; p = 0.538), suggesting that the weight regain trend between groups was no longer statistically distinguishable at the end of follow-up, though the direction of the estimate remained in favor of the IGB group, with an overall effect of -4.14% (95% CI: 7.06, -1.22%; p = 0.006) (Supplementary Figure S1 and Table S2).

The delta-adjusted pattern-mixture sensitivity analysis, conducted under a missing not at random (MNAR) framework with progressively conservative assumptions favoring the CBT group, demonstrated that the superior outcomes observed in the IGB group remained statistically significant at all three follow-up time points. Although the magnitude of the between-group differences attenuated modestly with delta adjustments, the direction and statistical significance of the treatment effect were preserved across all timepoints (Supplementary Table S2). These findings suggest that the primary results are robust to plausible departures from the missing at random assumption.
