# Supplementary material for: Intragastric Balloon Treatment Enhances Weight Maintenance Adjunct to Low‐Energy Diet and Group‐Based Cognitive Behavioural Therapy: A Randomized Controlled Trial
Source: Diabetes Obes Metab. 2026 Jun 3;28(8):7300–11. doi: 10.1111/dom.70865 (PMC13341412; doi:10.1111/dom.70865)
Supplement: Supplementary file 2 — Figure S2: Mean change in body weight (%) from TBW for both the main analysis as shown in Figure 1 and a sensitivity analysis including 44 of 51 participants (86.3%) in the IGB group who completed at least 6 months of IGB treatment and had available weight data at 18 months, and 43 of 56 participants (76.8%) in the CBT group with weight data at 18 months. [file DOM-28-7300-s005.docx]

**Supplementary Figure S2.** Mean change in body weight (%) from TBW for both the main analysis as shown in Figure 1 and a sensitivity analysis including 44 of 51 participants (86.3%) in the IGB group who completed at least 6 months of IGB treatment and had available weight data at 18 months, and 43 of 56 participants (76.8%) in the CBT group with weight data at 18 months.


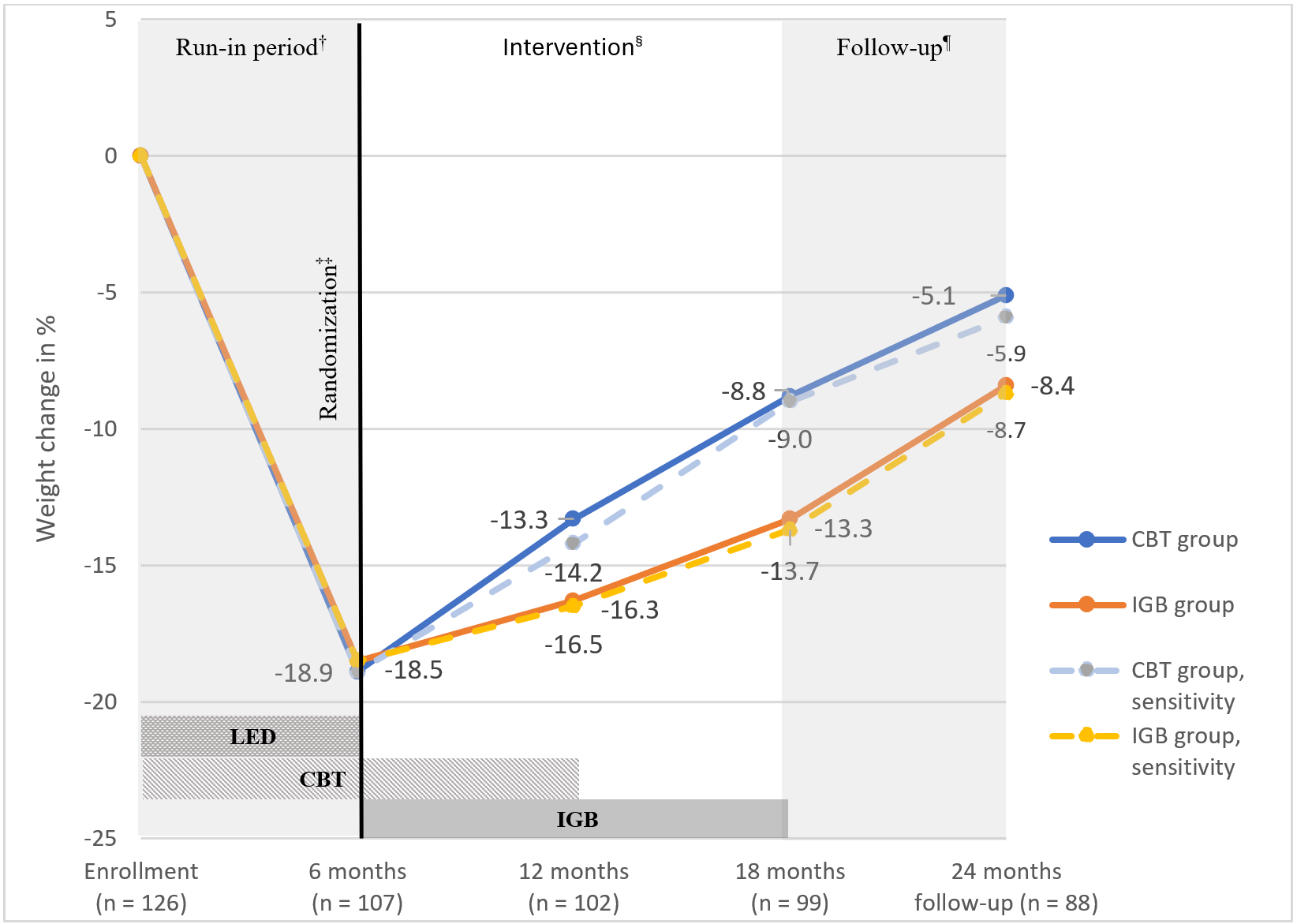


Abbreviations: BMI, body mass index; CBT (striped pattern), cognitive behavioral therapy; LED (checked pattern), low-energy diet; IGB (solid pattern), intragastric balloon; TBW, total body weight

† Run-in period; 6-month run-in period: all participants received a combination of group-based CBT and LED.

‡ Randomization; Participants were randomized, ratio 1:1, to either the IGB or CBT group.

§ Intervention; IGB group received 6 months CBT plus IGB treatment up to 12 months, CBT group received 6 months CBT.

¶ Follow-up; The 12-month treatment was followed by a 6-month follow-up period.
