# Supplementary material for: Intragastric Balloon Treatment Enhances Weight Maintenance Adjunct to Low‐Energy Diet and Group‐Based Cognitive Behavioural Therapy: A Randomized Controlled Trial
Source: Diabetes Obes Metab. 2026 Jun 3;28(8):7300–11. doi: 10.1111/dom.70865 (PMC13341412; doi:10.1111/dom.70865)
Supplement: Supplementary file 3 — Table S1: Inclusion and exclusion criteria. [file DOM-28-7300-s003.docx]

**Supplementary Table S1:** Inclusion and exclusion criteria

*Inclusion criteria*:

- BMI ≥32.5 and ≤45 kg/m²
- Age 30-65 years

*Exclusion criteria*:

- Organized weight reduction treatment within the last 3 months
- Daily use of meal replacement products within the last 3 months
- Previous gastric surgery
- Gastric, duodenal or oesophageal ulcers
- Inflammatory disease of the gastrointestinal tract
- Potential upper gastrointestinal bleeding conditions such as esophageal varices
- Current use of thrombocyte aggregation inhibitors and/or anticoagulants
- Structural abnormalities of the pharynx or esophagus
- Symptoms suggestive of severe gastric motility disorder such as persistent nausea and vomiting
- Hiatal hernia ≥5 cm
- Cancer diagnosed within the last 5 years or ongoing cancer treatment (except non-metastasizing skin cancer)
- Known severe heart failure (NYHA 3-4)
- Known chronic obstructive pulmonary disease (FEV_1_ ≤50%)
- Kidney failure (eGFR <30 ml/min)
- Liver failure or liver enzymes more than 3 times the normal threshold
- Known proliferative retinopathy
- Known or suspected alcohol or narcotics abuse
- Current or past systemic treatment with corticosteroids within the last 3 months
- Known myocardial infarction or stroke within the last 6 months
- Current or past history of pancreatitis
- Pregnancy, intention to become pregnant or breastfeeding during the study
- Untreated or insufficiently treated hypo- or hyperthyroidism
- Known or previous eating disorder
- Antimicrobial treatment within the last 3 months may lead to postponed participation.
- Regular consumption of probiotics within 1 month prior to study start may lead to postponed participation

Participants considered to be unsuitable for the study by the investigator (e.g. serious psychiatric disorders, suspected eating disorders)
