# Supplementary material for: Intragastric Balloon Treatment Enhances Weight Maintenance Adjunct to Low‐Energy Diet and Group‐Based Cognitive Behavioural Therapy: A Randomized Controlled Trial
Source: Diabetes Obes Metab. 2026 Jun 3;28(8):7300–11. doi: 10.1111/dom.70865 (PMC13341412; doi:10.1111/dom.70865)
Supplement: Supplementary file 4 — Table S2: Pooled treatment effects based on mixed effects models from 20 imputations under missing at random (MAR) and missing not at random (MNAR) assumptions. [file DOM-28-7300-s002.docx]

**Supplementary Table S2**: Pooled treatment effects based on mixed effects models from 20 imputations under missing at random (MAR) and missing not at random (MNAR) assumptions.

| **Comparison** | **MAR** | **MNAR (delta-adjusted)** |
| --- | --- | --- |
| IGB vs. CBT at 12 months | -3.33 (-5.50, -1.15); p = 0.003 | -3.24 (-5.42, -1.06); p = 0.004 |
| IGB vs. CBT at 18 months | -5.47 (-8.24, -2.70); p < 0.001 | -5.06 (-7.83, -2.29); p <0.001 |
| IGB vs. CBT at 24 months | -4.14 (-7.06, -1.22); p = 0.006 | -2.88 (-5.87, 0.12); p = 0.060 |
