# Supplementary material for: Intragastric Balloon Treatment Enhances Weight Maintenance Adjunct to Low‐Energy Diet and Group‐Based Cognitive Behavioural Therapy: A Randomized Controlled Trial
Source: Diabetes Obes Metab. 2026 Jun 3;28(8):7300–11. doi: 10.1111/dom.70865 (PMC13341412; doi:10.1111/dom.70865)
Supplement: Supplementary file 5 — Table S3: Adverse events from enrollment to 18 months. [file DOM-28-7300-s004.docx]

| Supplementary Table S3. Adverse Events from enrollment to 18 months | | | |
| --- | --- | --- | --- |
|  | ***6-month run-in***  ***(n = 126), No. of participants*** | ***Randomization period, 6- to 18-months after enrollment, No. of participants*** | |
|  |  | **IGB (n = 51)** | **CBT (n = 56)** |
| Adverse Events^†^ | | | |
| Gastrointestinal AE^‡^ |  | | |
| Nausea | 10 | 44 | 0 |
| Constipation | 30 | 19 | 1 |
| Stomach cramps | 10 | 40 | 0 |
| Vomiting | 0 | 36 | 0 |
| Loose stool | 23 | 10 | 0 |
| Bloating (stomach) | 4 | 22 | 1 |
| Dyspepsia | 11 | 11 | 3 |
| Excessive burping | 0 | 21 | 0 |
| Dizziness | 40 | 0 | 0 |
| Infection^§^ | 20 | 7 | 6 |
| Psychological symptoms | 8 | 6 | 8 |
| Fatigue | 19 | 0 | 0 |
| Hair loss | 16 | 0 | 0 |
| Mechanical symptoms of IGB, e.g. feeling its presence when moving or lying down | NA | 7 | NA |
| Symptomatic cholelithiasis | 2 | 1 | 3 |
| Headache | 6 | 0 | 0 |
| Excess skin after weight loss | 5 | 0 | 0 |
| Other (itching, dry mouth) | 4 | 0 | 0 |
| Musculoskeletal symptoms | 0 | 0 | 4 |
| Heart palpitations | 0 | 1 | 1 |
| Iron deficiency | 0 | 0 | 1 |
| Vasovagal collapse | 0 | 0 | 1 |
| Serious Adverse Events^†^ | | | |
| Appendectomy | 2 | 0 | 0 |
| Cancer | 0 | 0 | 1 |
| Cholecystectomy | 0 | 0 | 3 |
| Eating disorder (anorexia nervosa, bulimia nervosa) | 0 | 0 | 2 |
| Gastric ulcer | 1 | 0 | 0 |
| Hospital admission due to GI symptoms | 0 | 4 | 0 |
| Threatened airway during IGB extraction | NA | 3 | NA |
| Mortality | 0 | 0 | 0 |
| Total SAEs | 2 | 7 | 6 |

†: Based on both perceived adverse events and review of medical files. Participants were asked to report all possible adverse events at each visit and to indicate whether any medical treatment had been required, regardless of the reason. Adverse events were recorded if symptoms were perceived by the participant, regardless of whether treatment was necessary.

‡: Gastrointestinal symptoms were specifically assessed during the first week of IGB treatment via telephone calls (1 to 3 calls) in the IGB group. The CBT group was not contacted.

§: *Infections during run-in*: upper respiratory tract infection (6), COVID-19 (5), lower urinary tract infection (2), endometritis (2), mastitis (1), erysipelas (1), thrombophlebitis (1), bacterial vaginosis (1), pneumonia (1). *Infections during intervention-period:* *IGB group*: COVID-19 (2), lower urinary tract infection (1), herpes simplex (1), mastitis (1), gastroenteritis (1), ear infection (1). *CBT group*: COVID-19 (2), infected wound (2), upper respiratory tract infection (1), Borrelia infection (1).

Four participants discontinued within 1 month due to an AE/SAE: three due to nausea related to meal replacements and one due to appendicitis. Abbreviations: AE, adverse event; SAE, serious adverse event; CBT, cognitive behavioral therapy; GI, gastrointestinal; IGB, intragastric balloon; NA, not applicable
